# Early Independent Wheeled Mobility in Children with Cerebral Palsy: A Norwegian Population-Based Registry Study

**DOI:** 10.3390/jcm14030923

**Published:** 2025-01-30

**Authors:** Anne Kilde, Kari Anne I. Evensen, Nina Kløve, Elisabet Rodby-Bousquet, Stian Lydersen, Gunvor Lilleholt Klevberg

**Affiliations:** 1 Division of Pediatric and Adolescent Medicine, Akershus University Hospital, 1478 Lørenskog, Norway; anne.kilde@ahus.no; 2 Department of Rehabilitation Science and Health Technology, Oslo Metropolitan University, 0130 Oslo, Norway; karianne.i.evensen@ntnu.no; 3 Department of Clinical and Molecular Medicine, Norwegian University of Science and Technology, 7491 Trondheim, Norway; 4 Children’s Clinic, St. Olav’s University Hospital, 7030 Trondheim, Norway; 5 Department of Clinical Neuroscience for Children, Norwegian Quality and Surveillance Registry for Cerebral Palsy, Oslo University Hospital, 0424 Oslo, Norway; nikloe@ous-hf.no (N.K.); elisabet.rodby_bousquet@med.lu.se (E.R.-B.); 6 Department of Clinical Sciences, Orthopaedics, Lund University, SE-22100 Lund, Sweden; 7 Department of Mental Health, Norwegian University of Science and Technology, 7491 Trondheim, Norway; stian.lydersen@ntnu.no

**Keywords:** cerebral palsy, wheelchair, assistive device, independent mobility, powered mobility, early intervention

## Abstract

**Background**: The aim was to explore independent wheeled mobility in children with CP, and identify predictors of early independent wheeled mobility and changes over time across birth cohorts. **Methods**: We included data from the Norwegian Quality and Surveillance Registry for Cerebral Palsy (NorCP) comprising 11,565 assessments of 1780 children born in 2002–2019. Variables included demographic data, Gross Motor Function Classification System (GMFCS) and Manual Ability Classification System (MACS) levels, wheelchair use, and independent wheeled mobility. Cox proportional hazard regression was used to identify predictors for early independent wheeled mobility. Kaplan–Meier survival curves were used to compare birth cohorts. **Results**: Of 769 (43%) children who used a wheelchair, 511 (67%) had independent wheeled mobility. Two thirds of the children (*n* = 337) achieved independent wheeled mobility before age 7. Most children with independent wheeled mobility used powered wheelchairs. Children at GMFCS levels III and IV were more likely to reach independent wheeled mobility at an early age. Children at MACS levels III–V had a lower probability of early independent wheeled mobility. The average age of achieving independent mobility decreased from 9.5 to 4.0 years between birth years 2002 and 2019. **Conclusions**: Two in three children were independent wheelchair users before 7 years of age, and the age of obtaining independent wheeled mobility has decreased over the last 20 years. Children with better hand function were more likely to obtain early independent wheeled mobility. Early intervention programs to promote mobility, development and participation should include powered mobility, adapted steering options, and interventions for hand function.

## 1. Introduction

Independent mobility is a major developmental milestone with important implications for children’s socio-emotional development and parent–child interaction [1]. Adolescents with physical disabilities have identified being present where things happen as the single most important factor for participation [2]. This requires the ability to move around actively in different settings.

Independent mobility provides new opportunities to explore and engage in the environment, enables new experiences, and affords children the opportunity to exert some degree of control [3,4,5]. Children with mobility limitations, such as cerebral palsy (CP), require different mobility options to explore and navigate in their environment and to take advantage of opportunities for social interaction and participation [6,7,8]. Despite growing evidence of the impact of self-mobility on overall development [9,10], a gap still exists between current knowledge and clinical practice, especially when prescribing powered mobility devices to young children [11,12]. Despite reports that independent mobility is more often reached in powered versus manual wheelchairs, younger children with CP still have access mainly to manual wheelchairs [13]. Powered wheelchairs, ride-on toy cars, and other mobility devices allow children to initiate mobility, which is crucial to enable play, the development of psychosocial skills, interpersonal relationships, and general participation [8,11,14,15,16].

In recent years, the early detection and diagnosis of CP has improved. An accurate and timely diagnosis of CP facilitates the implementation of tailored early intervention programs, which can optimize a child’s brain plasticity, support their development, and improve caregiver well-being [17]. The complexity of motor, cognitive and visual challenges experienced by young children with CP can limit their functional mobility options. Consequently, a multidisciplinary approach has been recommended in the international clinical practice guideline for early intervention in children with CP [18]. Although the guideline does not specifically include recommendations for wheeled mobility, the authors emphasized the importance of the frequent practice of activities that facilitate skilled movement and functional independence. A recent randomized controlled trial investigating early powered mobility in young children with CP has provided evidence supporting the inclusion of early powered mobility as a strategy to enhance functional skills and promote independence [19]. This has also been supported by qualitative data wherein parents described how powered mobility enhanced their children’s independence and self-efficacy, providing the child with more opportunities to make independent choices and to object to choices made by parents [20]. To our knowledge, the extent to which wheeled mobility is introduced at early ages has not been previously examined in large population-based cohorts.

Only two population-based studies have previously described wheelchair use and independent wheeled mobility in children with CP [13,21]. Data from the Swedish national follow-up program for CP showed that children were less likely to self-propel manual wheelchairs compared to powered wheelchairs, regardless of age, gross motor function, or hand function, suggesting that powered mobility should be introduced at earlier ages to promote independent mobility [13]. In a mixed-methods review of the effectiveness of powered mobility for children, Bray et al. (2020) concluded that powered mobility for children under 5 years of age seems to have multiple benefits [22].

The purpose of this study was to explore independent wheeled mobility in children with CP according to age, motor function levels, and CP subtypes. Additionally, we aimed to identify factors associated with early independent wheeled mobility and changes in the age of independent wheeled mobility across birth cohorts from 2002 to 2019, using data from the Norwegian Quality and Surveillance Registry for Cerebral Palsy (NorCP). Our research questions were: (1) What are the proportions and characteristics of children who reach independent wheeled mobility, particularly below 7 years of age? (2) To what extent do CP subtype, gross motor function, manual ability and birth year predict early independent wheeled mobility? (3) Has the age for independent wheeled mobility decreased over the last 20 years?

## 2. Materials and Methods

### 2.1. Study Design

The study included longitudinal data registered in NorCP from January 2006 to October 2021. Data for birth years 2002–2006 included one health region (Helse Sør-Øst, covering approximately half of the country’s population), while data for children born 2007–2019 included approximately 90% of all Norwegian children with CP in these birth years [23]. The NorCP registry includes the classification of CP subtypes, levels of the Gross Motor Function Classification System (GMFCS) and the Manual Ability Classification System (MACS), mobility and use of wheelchairs, clinical assessments, and interventions [23]. Children are followed systematically and registered in NorCP annually or every other year by regional physiotherapists (PT) and occupational therapists (OT) from specialized healthcare sectors in close collaboration with their local therapists in the communities.

### 2.2. Participants

Data from 11,565 assessments of 1780 children registered in NorCP from January 2006 to October 2021 (birth years 2002–2019) were used to identify the total number of wheelchair users, independent wheelchair users, and age for independent wheeled mobility.

### 2.3. Variables and Data Collection

Data from NorCP assessments include information on use of manual or powered wheelchair for mobility and the ability to self-propel, obtained through the following questions posed to the child or caregiver: “Does the child use a wheelchair? (1) Manual wheelchair for mobility indoors? (2) Powered wheelchair for mobility indoors? (3) Manual wheelchair for mobility outdoors? (4) Powered wheelchair for mobility outdoors?” The response options were (a) yes, self-propels; (b) yes, is pushed/transported; (c) no wheelchair. Two new collapsed variables were created based on these questions, as follows: wheelchair use, including all who used wheelchairs in any setting; independent wheeled mobility, including those who self-propelled either a manual or a powered wheelchair.

CP subtypes were classified according to ICD-10 into hemiplegic, diplegic, quadriplegic, dyskinetic, ataxic, or unspecified. The GMFCS [24] level I to V was used to classify gross motor function. GMFCS is an age-related five-level classification system in which level I describes the most independent and level V the least independent levels of gross motor function. Manual ability was classified according to the five ordinal levels of MACS, ranging from level I, indicating ability to handle age-appropriate objects easily and successfully, to level V, indicating severely limited ability to handle even simple objects [25]. Age was grouped into four categories based on the age of first being registered as using a wheelchair or having independent wheeled mobility, as follows: 0–3 years, 4–6 years, 7–12 years, and 13–18 years. Four cohorts were created based on birth years—2002–2005, 2006–2010, 2011–2014, and 2015–2019.

### 2.4. Statistical Analyses

Descriptive statistics for age were presented with mean, standard deviation (SD), median, and min/max. Categorical data were presented as frequencies and percentages.

**Figure 1:**
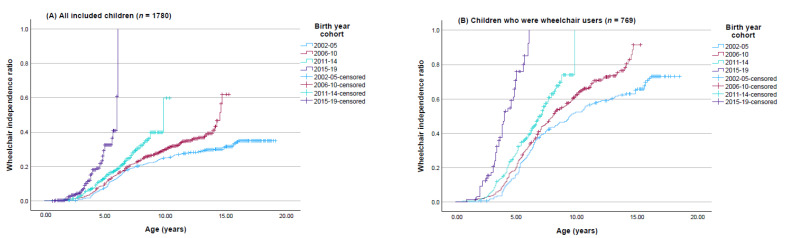
Kaplan–Meier survival curves for age of reaching independent wheeled mobility, according to four birth cohorts from 2002–2019. Estimated for (**A**) all included children, and (**B**) only children who were wheelchair users.

Cox proportional hazard regression analysis was used to identify predictors for earlier independent wheeled mobility. We use the term “independent wheelchair ratio” to reflect hazard ratio in this context. When this ratio is over (under) 1 for a given covariate, then the likelihood of earlier independent wheeled mobility increases (decreases) with this covariate. The following covariates were included one at a time (unadjusted), prior to including all covariates simultaneously (adjusted): sex, birth year cohort (4 categories), CP subtype (5 categories), GMFCS (5 categories), and MACS (5 categories). All children were included in the primary analyses, but children who were not wheelchair users were excluded from a secondary analysis. Kaplan–Meier survival plots [26] were used to show differences in time from birth to event (i.e., age of independent wheeled mobility) between the four birth cohorts (Figure 1). For some participants, the time from birth to the first report of independent wheeled mobility (i.e., age at the event) was not observed, and was censored at their last assessment. Table 1 shows the number of participants with different reasons for censoring. Children who were wheelchair users but dead (*n* = 17) or emigrated (*n* = 2) without reaching independent wheeled mobility were censored at the age of their last assessment. Children who were wheelchair users but with no report on independence status (*n* = 13) were excluded from the analysis. In the primary analysis, all children with valid data were included, yet children who were non-wheelchair users (*n* = 999) were censored at their last assessment. In the secondary analysis, all non-wheelchair users were excluded. Children with no registered data on wheelchair use (*n* = 12) were excluded from all analyses.

We report 95% confidence intervals (CI) where relevant and regard *p*-values under 0.05 to represent statistical significance. SPSS version 27.0 was used for the statistical analyses.

## 3. Results

### 3.1. Background Characteristics

Among the 1780 included children, there were more boys (58%) than girls (42%) (Table 2). Most children (87%) had spastic CP (including hemiplegia 44%, diplegia 30%, and quadriplegia 13%), whereas 7% had dyskinetic CP, 4% ataxic CP, and 2% unspecified CP. Approximately two thirds were classified at GMFCS levels I and II (69%) or MACS I and II (67%), while less than one third were classified at GMFCS levels III to V (30%) or MACS III to V (29%) (Table 2). The mean age at the first assessment was 45.4 (SD 33.5) months; median age was 36.0 (range 6–208) months. The mean age at the final assessment was 119.7 (SD 53.0) months, and median age was 123.0 (range 8–229) months.

### 3.2. Wheelchair Users

In total, 769 (43%) children used wheelchairs (Table 2), with 597 (78%) getting their first wheelchair before 7 years of age. Wheelchair users were distributed across all CP subtypes, GMFCS levels, and MACS levels. Most children with quadriplegic (86%) and dyskinetic (79%) CP subtypes used wheelchairs, compared with only 18% of children with hemiplegic CP (Table 2). The largest proportion of wheelchair users was classified at GMFCS level III (96%) and the smallest proportion at GMFCS level I (12%). For hand function, the largest proportion of wheelchair users was at MACS level V (90%) and the smallest at MACS level I (19%).

### 3.3. Independent Wheeled Mobility

Independent wheeled mobility was achieved by 511 children (67% of all of the wheelchair users) (Table 2). The largest proportions of children with independent wheeled mobility were found within the ataxic (88%) and diplegic (81%) CP subtypes, and almost half of the children with quadriplegic CP had independent wheeled mobility (48%). For gross motor function, the largest proportion of children with independent wheeled mobility was in GMFCS level III (86%), and the smallest proportion was at GMFCS level V (38%). For hand function, the largest proportion with independent wheeled mobility was at MACS level II (82%) and the smallest was at level V (37%).

Almost half of the children (46%) with independent wheeled mobility reached independence at between 4 and 6 years of age, and by the age of 7 years, two thirds of the children (*n* = 337, 66%) had achieved independent wheeled mobility (Table 3).

For all age groups except 0–3 years, most of the children with independent wheeled mobility used powered wheelchairs (Table 3). Among children under 7 years of age, 42% (*n* = 140) were independent in manual wheelchairs, while 45% (*n* = 151) were independent in powered wheelchairs. Some of the children in all age groups were independent users of both manual and powered wheelchairs (*n* = 53, 10%); data were missing on type of wheelchair for 27% (*n* = 137).

### 3.4. Predictors of Early Independent Wheeled Mobility

In the primary Cox regression analysis with all children included (*n* = 1766, missing data *n* = 14, Table 4), there was a significant (*p* < 0.001) increase in the likelihood of reaching earlier independent wheeled mobility for each consecutive birth cohort after 2002–2005. In the multiadjusted analysis, children born in 2015–2019 were more than four times more likely to be independent wheelchair users at an early age compared to children born in 2002–2005 (ratio 4.38, 95% CI 2.92–6.57, *p* < 0.001). Compared to children with hemiplegic CP, children with diplegic (ratio 1.45, 95% CI 1.08–1.95, *p* = 0.013) and ataxic (ratio 1.59, 95% CI 1.02–2.47, *p* = 0.039) CP were more likely to reach independent wheeled mobility at an early age. Children classified at GMFCS levels II–V were more likely to reach independent wheeled mobility at an early age compared to GMFCS level I, yet for the MACS levels, children classified at level IV (ratio 0.65, 95% CI 0.43–1.00, *p* = 0.052) and V (ratio 0.37, 95% CI 0.22–0.63, *p* < 0.001) were less likely to reach independent wheeled mobility at an early age compared to MACS level I.

In the secondary Cox regression analysis, including only wheelchair users (*n* = 769, Table 4), children born in 2015–2019 were almost six times as likely to reach early independent wheeled mobility as children born in 2002–2005 (ratio 5.74, 95% CI 3.83–8.59, *p* < 0.001); this difference was significant for all cohorts after 2002–2005. In the multiadjusted analysis, CP subtype was not a significant predictor of early independent wheeled mobility. Children classified at GMFCS levels III and IV were over two (ratio 2.69, 95% CI 1.86–3.90, *p* < 0.001) and three (ratio 3.62, 95% CI 2.36–5.57, *p* < 0.001) times as likely to reach independent wheeled mobility at an early age compared to level I, in contrast to children classified at GMFCS levels II (ratio 1.16, 95% CI 0.85–1.57, *p* = 0.36) and V (ratio 1.30, 95% CI 0.73–2.29, *p* = 0.38). Children classified at MACS levels III to V were less likely to reach independent wheeled mobility at an early age compared to MACS levels I, yet the difference was significant only for level IV (ratio 0.44, 95% CI 0.29–0.66, *p* < 0.001) and V (ratio 0.27, 95% CI 0.29 to 0.66, *p* < 0.001).

In accordance with the Cox regression analyses, the Kaplan–Meier survival curves (Figure 1) show the changes over time for age of independent wheeled mobility between the four birth cohorts. Estimated medians and quartiles for the age for each birth cohort are presented in Table 5. In both the primary and secondary analyses, independent wheeled mobility was reached at significantly younger ages for the most recent birth cohorts. The median age at independent wheeled mobility decreased significantly over time, from 9.5 years for children born 20 years ago (birth cohort 2002–2005) to 4 years for the most recent birth cohort.

## 4. Discussion

In this population-based registry study with data from 11,565 assessments for 1780 children with CP born from 2002 to 2019, two thirds of the wheelchair users had independent wheeled mobility. Two thirds of the children reached this independence before the age of 7 years, with the largest proportion between 4–6 years. For all age groups except 0–3 years, the majority of children with independent wheeled mobility used powered wheelchairs. The likelihood of reaching early independent wheeled mobility was positively associated with GMFCS levels III and IV and negatively associated with more limited hand function (MACS IV–V). Children born in the most recent birth years reached independent wheeled mobility at a significantly younger age compared to children born in the earlier birth years.

The strengths of this study include the use of registry data from NorCP with a large sample size. The NorCP covers >90% of the Norwegian population of children with CP [23]. For the earliest cohort, participants were included only from the health region of southeastern Norway. This region represents half of the country’s population, and the distribution of CP subtypes and functional levels is representative of the national CP population [27]. The NorCP webpage contains the NorCP protocols and corresponding manuals with definitions and descriptions; there is a network of local professionals (i.e., OTs, PTs, pediatricians, and psychologists) who participate in learning activities and discussions to ensure a common understanding of the included variables in the registry [28]. A limitation of the study is the relatively large number of missing data for independence in manual or powered wheelchairs, likely due to the non-mandatory nature of these variables in the registry. The study is population-based, and the results may be generalizable to other high-income countries with similar healthcare systems as in Scandinavia.

For 2016, national data on more than 2300 children registered in the Swedish CP follow-up program and quality registry (CPUP) show that, despite a small number of children using powered wheeled mobility, the proportion of children with independent mobility was much higher for those with powered wheelchairs compared to manual wheelchairs [13]. In our study, we have not reported on the proportion of manual versus powered wheelchair users, yet two thirds of the children who used a wheelchair reached independent wheeled mobility, and the largest proportion used powered wheelchairs.

Our results show that two thirds of the children with independent wheeled mobility became independent before 7 years of age, with the largest proportion (46%) learning to self-propel between 4 and 6 years of age. Among children both below and above 7 years of age, the largest proportion were independent in powered wheelchairs. More than half of the children aged 4–6, and one third of the children aged 0–3, were independent in powered wheelchairs. These results contrast with those from a study by Rodby-Bousquet and Hägglund (2010), wherein no children below 3 years of age were reported in the CPUP to use powered mobility [21]. Sweden and Norway are comparable Scandinavian countries, with similar healthcare systems based on a welfare model wherein all citizens have access to the same services, which are free of charge for children. The NorCP follow-up program is based on the Swedish CPUP [29]. A factor likely contributing to the difference in powered wheelchair use among the youngest children is the emergence of a wider selection of powered mobility devices suitable for small children during the 15 years since the data collection performed for the study by Rodby-Bousquet and Hägglund [21]. It may also reflect a significant gap between evidence-based recommendations and clinical practice in providing power mobility to young children, as previously reported from several countries [10,11,12].

In the primary Cox regression analysis, CP subtype was a highly significant predictor for early independent wheeled mobility for all CP subtypes compared to hemiplegia. In the secondary analysis, wherein only users of a wheelchair were included, children with diplegia and ataxia were more likely, and children with quadriplegia were less likely, to be early independent wheelchair users, compared to those with hemiplegia. In the adjusted model, only ataxia was a nearly significant predictor. To understand the clinical relevance of these findings, we need to look at the functional profiles of the CP subtypes as described by Patel et al. [30], Arner et al. [31], and Andersen et al. [32]. Whereas children with hemiplegic CP most often walk independently (GMFCS I–III) and handle objects in daily life independently (MACS I–II), children with diplegic CP are found within all GMFCS levels, yet most often in MACS levels I–II. Children with ataxic CP most frequently are classified at GMFCS levels I–II, and despite hand function classified within the whole functional range (MACS I–V), they most often handle objects independently (MACS I–II) and do not have the asymmetry that makes the self-propelling of manual wheelchair particularly difficult for those with hemiplegic CP.

In addition to predicting independent wheeled mobility, as shown in previous research [13], this study shows that GMFCS and MACS levels also predict the likelihood of independent wheeled mobility at an early age. Children classified at GMFCS III–IV were most likely to be early independent users of a wheelchair compared to GMFCS level I. Moreover, children classified at GMFCS level V were almost equally likely to reach early independent wheeled mobility as children at GMFCS level I, a finding that needs some reflection. The included wheelchair variables represent whether the person uses a wheelchair and whether it self-propels (performance), not whether it is able to self-propel (capacity). By definition, a child classified at GMFCS level I “walks without limitations” [33], and does not need a wheelchair for mobility per se. Nevertheless, even ambulant children with CP experience more fatigue than the typically developing population [34,35]; hence those classified at GMFCS level I may use their wheelchairs merely to save energy despite the capacity to use the wheelchair independently. On the other hand, GMFCS level V represents a group of children with complex disability, defined as “transported in manual wheelchair” [33], and often classified at MACS levels IV or V [36,37], which reduces the probability of independent wheeled mobility.

The finding that MACS levels III–V reduce the likelihood of early independent wheeled mobility has important clinical implications. There is a close association between MACS level V and GMFCS level V [36]. In line with our results, and as per the extended definition, children classified at GMFCS level V “can achieve self-mobility only if they learn how to operate a powered wheelchair” [24]. Increasing the opportunities for independent wheeled mobility among these children requires the thorough assessment of the individual child’s most precise and goal-directed movements, and of possibilities to adapt the operating system (joystick) accordingly, such as control by eye-gaze or head array [38].

For all age cohorts except 0–3 years, the use of powered wheelchairs increased the probability of independent wheeled mobility. More than half of the children with independent wheeled mobility at 4–6 years of age used a powered wheelchair, while 39% were independent in manual wheelchairs. Even children below 4 years of age reached independence in powered wheelchairs. This finding challenges the clinical tradition of introducing manual wheelchairs first and transitioning to powered wheelchairs later. Although many therapists may hesitate to introduce a powered wheelchair to a young child who may have the potential to achieve some walking ability, our results indicate a promising tendency toward earlier introduction to powered mobility. The results show an almost six times higher likelihood of early wheeled mobility for birth years 2015–2019 compared to 2002–2005; compared to children born earlier, a higher proportion of children in the most recent birth years become independent users of powered mobility.

Being able to move independently around home, in kindergarten, or in the community represents an important milestone for all young children, and is an important concern for parents [39]. Numerous studies support the importance of early mobility for psychosocial development and play [5,40], for spatial perception [41], and for participation in everyday life [42]. It is encouraging to find that the proportion of young children who self-propel their wheelchairs has increased over the last 20 years. This trend probably results from better access to powered mobility devices for young children and improved technologies for alternate operation modes to compensate for limited hand function, and also from a shift in the therapeutic view of independent wheeled mobility being not just for transportation, but also a facilitator for overall development and general participation. Although the international clinical practice guidelines for early intervention in children with CP does not include specific recommendations for early wheeled mobility [18], several studies have reported on the multiple benefits of such interventions [7,8,14,19,22]. More research is still needed to strengthen the knowledge base so as to support early wheeled mobility as a standard practice in early intervention programs. Meanwhile, policy arrangements should reflect our findings together with the current knowledge, and wheeled mobility devices should be accepted as useful tools to stimulate overall development not only for children with the most limited walking ability.

## 5. Conclusions

In this large population-based registry study, two out of three children with CP who used wheelchairs moved around independently, and a majority with independent wheeled mobility used powered wheelchairs. Children classified at GMFCS levels III–IV were most likely to be independent wheelchair users at an early age. Children with more limited hand function were less likely to be early independent wheelchair users. Advances in technology over the last 20 years, and a shift in the therapeutic views of wheeled mobility as a facilitator of development, have likely contributed to the significant decrease in the age of independent wheeled mobility. To promote early mobility, general development, and participation, early intervention programs for young children with CP should include powered mobility, adapted steering options, and interventions to promote hand function. 

## Figures and Tables

**Table 1:** Different sequences in the data set (N = 1780).

			Age at Independent Wheeled Mobility	
		*n*	Primary Analysis	Secondary Analysis
Wheelchair user		769		
	Independent	511	Known	Known
	Non-independent/dead	17	Censored	Censored
	Non-independent/emigrated	2	Censored	Censored
	Non-independent	226	Censored	Censored
	Unknown independence status	13	Excluded	Excluded
Non-wheelchair user		999		
	GMFCS I–II	943	Censored	Excluded
	GMFCS III–V	56	Censored	Excluded
Missing data for wheelchair use		12	Excluded	Excluded

Note: GMFCS—Gross Motor Classification System.

**Table 2:** The descriptive statistics for all 1780 children with cerebral palsy (birth years 2002–2019), children who used wheelchairs (assisted or independent), children with independent wheeled mobility (any type), and children with independent wheeled mobility in a manual or powered wheelchair.

		Total	Wheelchair User	Independent Wheeled Mobility	Independent Manual Wheelchair	Independent Powered Wheelchair	Independent Wheelchair Both	Independent Wheelchair Unknown
		N (%)	N (%) ^1^	N (%) ^2^	N (%) ^3^	N (%) ^3^	N (%) ^3^	N (%) ^3^
**Total number**		1780	769 (43.1)	511 (66.5)	196 (38.4)	237 (46.4)	53 (10.4)	137 (26.8)
**Sex**								
Girls		744 (41.8)	320 (43.0)	220 (68.8)	83 (37.7)	95 (43.2)	24 (10.9)	67 (30.5)
Boys		1036 (58.2)	449 (43.3)	291 (64.8)	113 (38.8)	142 (48.8)	29 (10.0)	70 (24.1)
**Birth year**								
2002–2005		367 (20.6)	175 (47.7)	118 (67.4)	46 (38.9)	51 (43.2)	12 (10.2)	36 (30.5)
2006–2010		704 (39.6)	335 (47.6)	237 (70.7)	88 (37.1)	101 (42.6)	20 (8.4)	68 (28.7)
2011–2014		419 (23.5)	192 (45.8)	111 (57.8)	47 (42.3)	55 (49.5)	15 (13.5)	24 (21.6)
2015–2019		290 (16.3)	67 (23.1)	45 (67.2)	15 (33.3)	30 (66.7)	6 (13.3)	9 (20.0)
**CP subtype**								
Spastic hemiplegia		785 (44.1)	139 (17.7)	95 (68.3)	21 (22.1)	60 (63.2)	7 (7.4)	22 (23.2)
Spastic diplegia		535 (30.1)	280 (52.3)	228 (81.4)	125 (54.8)	119 (52.2)	35 (15.4)	24 (10.5)
Spastic quadriplegia		231 (13.0)	198 (85.7)	94 (47.5)	14 (14.9)	18 (19.1)	4 (4.3)	66 (70.2)
Dyskinetic		130 (7.3)	103 (79.2)	59 (57.3)	19 (32.2)	26 (44.1)	5 (8.5)	19 (32.2)
Ataxic		69 (3.9)	32 (46.4)	28 (87.5)	13 (46.4)	13 (46.4)	2 (7.1)	4 (14.3)
Unspecified		30 (1.7)	17 (56.7)	7 (41.2)	4 (57.1)	1 (14.3)	0 (0)	0 (0)
**GMFCS**								
I		943 (53.0)	117 (12.4)	80 (68.4)	17 (21.3)	56 (70.0)	7 (8.8)	14 (17.5)
II		276 (15.5)	162 (58.7)	123 (75.9)	46 (37.4)	78 (63.4)	18 (14.6)	19 (15.4)
III		135 (7.6)	129 (95.6)	111 (86.0)	75 (67.6)	46 (41.4)	14 (12.6)	8 (7.2)
IV		161 (9.0)	138 (85.7)	113 (81.9)	55 (48.7)	46 (40.7)	13 (11.5)	25 (22.1)
V		253 (14.2)	221 (87.4)	84 (38.0)	3 (3.6)	11 (13.1)	1 (1.2)	71 (84.5)
Missing		12 (0.7)	2 (16.7)	0 (0)	0 (0)	0 (0)	0 (0)	0 (0)
**MACS**								
I		719 (40.4)	136 (18.9)	104 (76.5)	49 (47.1)	60 (57.7)	16 (15.4)	14 (13.5)
II		468 (26.3)	178 (38.0)	145 (81.5)	70 (48.3)	83 (57.2)	22 (15.7)	14 (9.7)
III		202 (11.3)	139 (68.8)	108 (77.7)	48 (44.4)	49 (45.4)	7 (6.5)	21 (19.4)
IV		119 (6.7)	106 (89.1)	72 (67.9)	24 (33.3)	29 (40.3)	6 (8.3)	25 (34.7)
V		211 (11.9)	189 (89.6)	70 (37.0)	2 (2.9)	8 (66.7)	1 (1.4)	61 (22.9)
Missing		61 (3.4)	21 (34.4)	12 (57.1)	3 (25.0)	8 (66.7)	0 (0)	0 (0)

CP: Cerebral palsy. GMFCS: Gross Motor Classification System. MACS: Manual Ability Classification System. ^1^ Percentages by row, from total number of children registered in each category. ^2^ Percentages by row, from number of children who were wheelchair users in each category. ^3^ Percentages by row, from number of children with independent wheeled mobility in each category.

**Table 3:** Distribution of children with independent wheeled mobility in manual or powered wheelchair, according to the age when first registered with independent wheeled mobility.

		Total *n* (%)	Independent Manual Wheelchair	Independent Powered Wheelchair	Independent Both Wheelchair Types	Independent Unknown Wheelchair Type
Total, *n* (%)		511	196 (38.4) ^1^	237 (46.4) ^1^	53 (10.4) ^1^	137 (26.8) ^1^
Age						
0–6 years		337 (65.9) ^1^	140 (41.5) ^2^	151 (44.8) ^2^	32 (9.4) ^2^	81 (24.0) ^2^
	0–3	101 (19.8) ^1^	49 (48.5) ^2^	27 (26.7) ^2^	6 (5.9) ^2^	34 (33.7) ^2^
	4–6	236 (46.2) ^1^	91 (38.6) ^2^	124 (52.5) ^2^	26 (11.0) ^2^	47 (19.9) ^2^
7–18 years		174 (34.1) ^1^	56 (32.2) ^2^	86 (49.4) ^2^	21 (12.1) ^2^	56 (32.2) ^2^
	7–12	150 (29.4) ^1^	51 (34.0) ^2^	79 (52.7) ^2^	20 (13.3) ^2^	41 (27.3) ^2^
	13–18	24 (4.7) ^1^	5 (20.8) ^2^	7 (28.0) ^2^	1 (4.2) ^2^	15 (62.5) ^2^

Note: ^1^ Percentages from total number of children (*n* = 511). ^2^ Percentages from total number of children for each row. For all age groups, some children were independent in both manual and powered wheelchairs.

**Table 4:** Cox proportional hazard regression analysis with age at independent wheeled mobility as the dependent variable, calculated for (A) primary analysis with all included children, and (B) secondary analysis with only children using a wheelchair. Unadjusted—with one covariate at a time; Adjusted—with all the listed covariates included.

	Unadjusted				Adjusted			
Covariate	*n*	Wheelchair Independence Ratio	95% CI	*p*	*n*	Wheelchair Independence Ratio	95% CI	*p*
**(A) Primary analysis**								
Total	511 ^1^/1766 ^2^				499 ^1^/1691 ^2^			
**Sex**								
boy	291/1028	ref			280/982	ref		
girl	220/738	1.01	0.85 to 1.20	0.90	219/709	1.16	0.97 to 1.39	0.11
**Birth year cohort**								
2002–2005	118/363	ref			116/360	ref		
2006–2010	237/697	1.43	1.13 to 1.81	0.003	234/687	1.61	1.27 to 2.03	<0.001
2011–2014	111/419	1.90	1.44 to 2.52	<0.001	110/406	1.73	1.30 to 2.31	<0.001
2015–2019	45/287	4.64	3.17 to 6.80	<0.001	39/238	4.38	2.92 to 6.57	<0.001
**CP subtype**								
Hemiplegia	95/779	ref			94/757	ref		
Diplegia	228/537	4.17	3.28 to 5.30	<0.001	221/512	1.45	1.08 to 1.95	0.013
Quadriplegia	94/222	3.88	2.92 to 5.16	<0.001	93/213	1.51	0.97 to 2.35	0.070
Dyskinetic	59/126	5.04	3.64 to 6.98	<0.001	57/118	1.34	0.89 to 2.03	0.17
Ataxic	28/68	4.10	2.69 to 6.26	<0.001	28/65	1.59	1.02 to 2.47	0.039
Unclassified	7/34	3.84	1.78 to 8.29	<0.001	6/26	1.30	0.55 to 3.04	0.55
**GMFCS**								
I	80/932	ref			79/912	ref		
II	123/276	6.41	4.83 to 8.49	<0.001	121/271	5.32	3.88 to 7.29	<0.001
III	111/135	24.97	18.63 to 33.46	<0.001	106/126	18.75	12.83 to 27.39	<0.001
IV	113/158	19.93	14.88 to 26.67	<0.001	109/148	23.24	14.98 to 36.05	<0.001
V	84/243	4.56	3.36 to 6.19	<0.001	84/234	9.75	5.50 to 17.28	<0.001
**MACS**								
I	104/713	ref			104/708	ref		
II	145/467	2.50	1.94 to 3.21	<0.001	145/462	1.36	1.04 to 1.79	0.026
III	108/200	5.38	4.10 to 7.04	<0.001	108/167	1.22	0.89 to 1.67	0.22
IV	72/120	5.96	4.41 to 8.06	<0.001	72/119	0.65	0.43 to 1.00	0.052
V	70/206	2.49	1.84 to 3.37	<0.001	70/205	0.37	0.22 to 0.63	<0.001
**(B) Secondary analysis**								
Total	511 ^1^/767 ^3^				499 ^1^/747 ^3^			
**Sex**								
boy	291/447	ref			280/431	ref		
girl	220/320	1.09	0.91 to 1.30	0.34	219/316	1.052	0.88 to 1.26	0.58
**Birth year cohort**								
2002–2005	118/175	ref			116/172	ref		
2006–2010	237/334	1.46	1.16 to 1.84	0.002	234/330	1.44	1.14 to 1.83	0.003
2011–2014	111/192	1.97	1.49 to 2.61	<0.001	110/189	1.75	1.32 to 2.33	<0.001
2015–2019	45/66	7.64	5.23 to 11.16	<0.001	39/56	5.74	3.83 to 8.59	<0.001
**CP subtype**								
Hemiplegia	95/138	ref			94/136	ref		
Diplegia	228/285	1.59	1.25 to 2.02	<0.001	221/276	1.07	0.79 to 1.44	0.68
Quadriplegia	94/198	0.61	0.46 to 0.81	<0.001	93/193	1.29	0.81 to 2.04	0.28
Dyskinetic	59/101	0.91	0.66 to 1.26	0.57	57/99	1.10	0.72 to 1.68	0.66
Ataxic	28/32	1.89	1.24 to 2.89	0.003	28/32	1.54	0.99 to 2.39	0.053
Unclassified	7/13	1.49	0.69 to 3.22	0.31	6/11	1.25	0.52 to 3.02	0.61
**GMFCS**								
I	80/116	ref			79/115	ref		
II	123/170	1.12	0.85 to 1.49	0.42	121/167	1.15	0.85 to 1.57	0.38
III	111/123	2.68	2.00 to 3.57	<0.001	106/116	2.68	1.86 to 3.90	<0.001
IV	113/141	2.09	1.57 to 2.79	<0.001	109/136	3.60	2.36 to 5.57	<0.001
V	84/216	0.45	0.33 to 0.61	<0.001	84/213	1.28	0.73 to 2.29	0.40
**MACS**								
I	104/136	ref			104/136	ref		
II	145/180	1.21	0.94 to 1.56	0.14	145/180	1.07	0.82 to 1.39	0.64
III	108/139	1.29	0.98 to 1.69	0.065	108/138	0.85	0.63 to 1.15	0.30
IV	72/106	0.89	0.66 to 1.21	0.45	72/106	0.44	0.29 to 0.66	<0.001
V	70/187	0.35	0.26 to 0.47	<0.001	70/187	0.27	0.16 to 0.44	<0.001

GMFCS: Gross Motor Function Classification System. MACS: Manual Ability Classification System. ^1^ Number of children with independent wheeled mobility. ^2^ Number of included children in the primary analysis. ^3^ Number of children who were wheelchair users.

**Table 5:** Estimated medians and quartiles for age (years) when reaching independent wheeled mobility among children in four birth cohorts, based on Kaplan–Meier survival analysis. Calculated for all the included children (primary analysis) and for children registered as wheelchair users (secondary analysis).

	Birth Cohort			
	2002–2005	2006–2010	2011–2014	2015–2019
	Median (Quartiles)	Median (Quartiles)	Median (Quartiles)	Median (Quartiles)
**Kaplan–Meier** **Estimate primary ^1^**	n.a. (9.9 to n.a.)	14.5 (8.3 to n.a.)	9.8 (7.1 to n.a.)	6.0 (4.8 to 6.1)
**Kaplan–Meier** **Estimate secondary ^2^**	9.5 (5.7 to n.a.)	8.0 (5.3 to 13.3)	7.1 (4.8 to 9.8)	4.0 (3.3 to 5.0)

n.a.: not available, cannot be computed because less than 50% (or 75%) in the cohort reached independent wheeled mobility. ^1^ Primary analysis. Based on all 1780 included children (*n* = 14 missing). LogRank: χ^2^ 71.954, df. 3, *p* < 0.001. ^2^ Secondary analysis. Based on 767 included children who were wheelchair users. LogRank: χ^2^ 141.202, df. 3, *p* < 0.001.

## Data Availability

The datasets analyzed during the current study are part of the NorCP registry and are not publicly available due to data protection regulations. The data are available from the corresponding author (G.L.K.) upon reasonable request.

## Abbreviations

CI	Confidence intervals
CP	Cerebral Palsy
GMFCS	Gross Motor Function Classification System
MACS	Manual Ability Classification System
OT	Occupational therapist
PT	Physiotherapist
NorCP	Norwegian Quality and Surveillance Registry for Cerebral Palsy
